# Editorial: Meiosis in plants: sexual reproduction, genetic variation and crop improvement

**DOI:** 10.3389/fpls.2023.1294591

**Published:** 2023-09-28

**Authors:** Yingxiang Wang, Changbin Chen, Gregory P. Copenhaver, Chung-Ju Rachel Wang

**Affiliations:** ^1^ College of Life Sciences, South China Agricultural University, Guangzhou, China; ^2^ Guangdong Laboratory for Lingnan Modern Agriculture, Guangzhou, China; ^3^ School of Life Sciences, Arizona State University, Tempe, AZ, United States; ^4^ Department of Biology and the Integrative Program for Biological and Genome Sciences, University of North Carolina at Chapel Hill, Chapel Hill, NC, United States; ^5^ Lineberger Comprehensive Cancer Center, University of North Carolina School of Medicine, Chapel Hill, NC, United States; ^6^ Institute of Plant and Microbial Biology, Academia Sinica, Taipei, Taiwan

**Keywords:** meiotic recombination, allopolyploid, double-strand-break, crossover, DMC1, PRD2, ZIP4, *FIGNL1*

Meiosis is essential for sexual reproduction and required for the formation of sperm and egg; its central events are the associations between homologous chromosomes (homologs), including pairing, synapsis, recombination and segregation. During recombination, the exchange of DNA between homologs results in new allelic combinations between the parents and offspring and among individual progeny (Wang and Copenhaver, 2018). This genetic variation is the foundation for biodiversity and speciation. The phenotypic diversity that results from genetic variation is also used to develop new elite traits during commercial plant and animal breeding practices. Thus, understanding the molecular mechanisms drive and regulate plant meiosis can accelerate crop improvement, and provide a theoretical foundation for the development and maintenance of new agricultural varieties.

Over the past three decades, molecular genetic studies have made great advances in understanding meiosis in plant species mainly by focusing on Arabidopsis, maize and rice. To improve our understanding of the potential applications of meiosis research in non-model plant or crop species, especially in polyploid plants, we organized this Research Topic which features seven original research articles covering five species including rice, wheat, allohexaploid Brassica, tomato, and *Elymus*. To present these findings in an accessible manner, we have organized them in to three themes: i) Molecular mechanism of meiotic recombination, ii) Meiosis in polyploid plants, and iii) Meiotic restitution induced by environmental stressors.

## Molecular mechanism of meiotic recombination

Our Research Focus includes two papers that advance our understanding of the mechanisms that govern double-strand-break (DSB) and crossover (CO) formation. Meiotic recombination is initiated by the formation of DSBs that require multiple proteins including MEIOSIS INHIBITOR 4 (MEI4/REC24/PRD2) (Cole et al., 2010). PRD2 was first demonstrated to be required for plant meiotic DSB formation in Arabidopsis (De Muyt et al., 2009). Wang et al. identified rice PRD2 and demonstrated that it plays a conserved role in meiotic DSB formation in monocots (rice) and dicots (Arabidopsis), which diverged 140-150 Myr ago (Chaw et al., 2004). This study also provided evidence that OsPRD2 is dispensable for spindle assembly and organization, which is different from the previous finding in Arabidopsis.

*FIDGETIN-LIKE-1* (*FIGNL1*) encodes a conserved AAA-ATPase that belongs to the FIDGETIN subfamily and acts later in recombination (Yuan and Chen, 2013). Arabidopsis has a single copy of FIGL1 that acts as an anti-crossover factor that limits class II CO formation, likely by influencing DMC1-mediated single strand invasion (Fernandes et al., 2018). Yang et al. identified the rice homolog FIGNL1 and demonstrated that, in addition to limiting class II CO formation, FIGNL1 is also required for limiting non-homologous chromosome associations during meiotic DSB repair. Moreover, FIGNL1 interacts with MEICA1 (meiotic chromosome association 1), which has a domain of unknown function (DUF4487). The chromosomal localization of FIGNL1 is dependent on synaptonemal complex assembly. Studying both of these the recombination-associated genes in rice has advanced our understanding of meiotic progression in plants, revealing both commonalities and differences.

## Meiosis in polyploid plants

Our Research Topic includes four papers that examine mechanisms that regulate meiosis in allopolyploid plants. Polyploidization, or the duplication of whole chromosome sets, is a very common phenomenon in plants. The widespread presence of polyploidy across plant taxa is counterintuitive since multi-chromosomal interaction between homologous and homeologous chromosomes poses a risk to proper chromosome segregation. Hexaploid wheat (bread wheat, *Triticum aestivum*) has been an important experimental system for exploring this issue because of *Pairing homeologous1 (Ph1)*, a complex locus that safeguards proper synapsis and crossing over between homologs (Sears, 1976). In *T. aestivum* the *ZIP4* paralogue *TaZIP4-B2* is located in the *Ph1* locus and promotes CO formation between homologs by suppressing COs between homeologous chromosomes (Rey et al., 2017). Draeger et al. reported that three *ZIP4* copies (*TtZIP4-A1, TtZIP4-B1* and *TtZIP4-B2*) exist in Tetraploid wheat (pasta wheat, *Triticum turgidum* ssp. *durum*), and that disruption of both *TtZIP4-A1/B1* genes results in a 76-78% reduction in COs, while mutation of all three genes leads to 95% reduction in COs, indicating that TtZIP4s are required for both Class I and II CO formation. In addition, *TtZIP4-B2* was found to have a strong effect on synapsis in wheat, which is distinct to the previous findings of ZIP4 in *Arabidopsis* and rice.

DMC1 is a meiosis-specific recombinase with weak intrinsic ATPase activity, which is conserved in most eukaryotes (Xu et al., 2023). Interestingly, Draeger et al. found that, in the hexaploid wheat variety ‘Chinese Spring’, a mutation of the D-genome homeolog of *DMC1* (*TaDMC1-D1*) causes a significant reduction of COs in a temperature responsive manner, exhibiting a more substantial change at low temperatures (13°C) compared with high temperatures (30°C). This finding not only reveals new information about how DMC1 is regulated in different species, but may also provide an important tool for improving wheat breeding and engineering resilience to climate change.

Artificially synthesized Brassica allohexaploids have superior stress resistance and high oilseed yield, but suffer from reduced fertility. Tian et al. used cytology and transcriptomics to investigate meiosis and pollen development in synthetic Brassica allohexaploids. Their results show that anther structure and tapetum development appears normal, but meiotic chromosome segregation is aberrant. Transcriptomic analyses showed that genes related to chromosome segregation were obviously downregulated in the synthetic Brassica allohexaploids, providing a potential explanation for the abnormal chromosome segregation.

*Elymus nutans*, a perennial grass and potential forage crop (Liu et al., 2022), is an allopolyploid with a StStYYHH (2n = 6x = 42) genome and is widely distributed in the Qinghai-Tibet Plateau of China. Populations of *E. nutans* have high levels of intergenomic translocations and chromosomal variations (Dou et al., 2017). As a result, *E. nutans* individuals that are heterozygous for chromosomal rearrangements often have meiotic defects. Liu et al. performed sequential fluorescence *in situ* hybridization (FISH) and genomic *in situ* hybridization (GISH) to examine the intra- and inter-genome chromosomal variations in *E. nutans* heterozytous plants. They observed that the meiotic chromosome abnormalities including bridges, fragments, unequal segregation, and lagging chromatids at various stages. In addition, this study also identified several paracentric inversions. This work highlights the utility of *E. nutans* as an important system for understanding chromosome structural variation in polyploid plants.

## Meiotic restitution induced by environmental stressors

Rounding out our Research Focus, we have a study on the influence of heat stress on meiosis in tomato. Meiotic restitution occurs when one of the two divisions of meiosis is disrupted resulting in the formation of diploid spores. Meiotic restitution can result from genetic variants, but can also be influenced by external factors such as ambient temperature as has been observed in rose, *Populus pseudosimonii*, and Arabidopsis (De Storme and Geelen, 2013). Other environmental factors including drought, and high-UV have also been implicated in triggering abnormal meiosis, and meiotic restitution (Qi and Zhang, 2019). Schindfessel et al. reported that, in tomato, heat stress (>35°C) interferes with homologous chromosome synapsis, thus leading to the defects in meiotic recombination and chromosome segregation. Interestingly, they also find that heat stress has a role in converting meiotic divisions to “mitotic-like” divisions, a form of restitution, likely due to influence the spindle organization, which results in the formation of diploid pollen.

In summary, this Research Topic brings together recent findings, highlighting multiple non-model crops to improve our understanding of the molecular mechanisms of plant meiosis, and provide new insights into how meiosis works in polyploidy plants. These advances will be useful for efforts to engineer meiosis and recombination to enhance agriculture.

## Author contributions

YW: Writing – original draft, Writing – review & editing. CC: Writing – original draft. GC: Writing – review & editing. CW: Writing – review & editing.
